# Publisher Correction: The globus pallidus orchestrates abnormal network dynamics in a model of Parkinsonism

**DOI:** 10.1038/s41467-020-15947-w

**Published:** 2020-04-16

**Authors:** Brice de la Crompe, Asier Aristieta, Arthur Leblois, Salma Elsherbiny, Thomas Boraud, Nicolas P. Mallet

**Affiliations:** 1 Université de Bordeaux, Institut des Maladies Neurodégénératives, 33076 Bordeaux, France; 2 CNRS UMR 5293, Institut des Maladies Neurodégénératives, 33076 Bordeaux, France

**Keywords:** Neuroscience, Neural circuits

Correction to: *Nature Communications* 10.1038/s41467-020-15352-3, published online 26 March 2020.

The original version of this Article contained an error in the Discussion, which incorrectly read ‘Striatal indirect neurons become selectively entrained during β-oscillations^40^ with a phase preference that precede STN activity by 45° (which corresponds to ~6 s assuming a 50 ms β-cycle period)^24^’. The correct version states ‘ms’ in place of ‘s’.

This has been corrected in both the PDF and HTML versions of the Article.

