# Supplementary material for: The globus pallidus orchestrates abnormal network dynamics in a model of Parkinsonism
Source: Nat Commun. 2020 Mar 26;11:1570. doi: 10.1038/s41467-020-15352-3 (PMC7099038; doi:10.1038/s41467-020-15352-3)
Supplement: Supplementary file 1 — Supplementary Information [file 41467_2020_15352_MOESM1_ESM.pdf]

The globus pallidus orchestrates abnormal network dynamics in Parkinsonism

Authors: Brice de la Crompe[1,2], Asier Aristieta[1,2], Arthur Leblois[1,2], Salma Elsherbiny[1,2], Thomas Boraud[1,2], Nicolas P. Mallet [1,2*]

[*]Correspondence should be addressed to nicolas.mallet@u-bordeaux.fr

Supplement Information files

Supplementary data (6 Supplementary Tables, 10 Supplementary Figures)

Supplementary Table 1. Relates to Figure 1

Figure	Parameter	n (rats/neurons)	Data Type	Data Value	Statistical test	significance level
**1e**	Firing Rate	5 /43	OFF	2.8 ± 0.6	Wilcoxon signed rank test	z=-5.23, *p*<0.001
			ON	0.7 ± 0.3		
**1i**	Firing Rate	8/30	OFF	27.3 ± 2.3	Wilcoxon signed rank test	z=-4.70, *p*<0.001
			ON	21.7 ± 2.2	Mann-Whitney Rank Sum Test	U=235, *p*<0.001
		15/68	Control	7.0 ± 0.7		
**1l**	*β*-AUC power	8/8 rec.	OFF	1.7 ± 0.1 $x10^{-1}$	paired *t*-test	t=1.69, *p*=0.135
			ON	1.6 ± 0.2 $x10^{-1}$		
**1n**	*β*-AUC power	8/6893 opto stim	Pre-OFF	1.1 ± 0.8 $x10^{-3}$	Wilcoxon signed rank test	z=--1.53, *p*=0.126
			OFF-ON	3.0 ± 0.8 $x10^{-3}$		
**1o**	*β*-phase locking	8/30	OFF	270.6 ± 6.5 °	Watson-Williams F test	F=1.42, *p*=0.240
			ON	284.5 ± 9.2 °		

Supplementary Table 2. Relates to Figure 2

Figure	Parameter	n (rats/neurons)	Data Type	Data Value	Statistical test	significance level
**2e**, CaMKII	Firing Rate	4/57	OFF	16.0 ± 1.4	Wilcoxon signed rank test	z=-4.46, *p*<0.001
			ON	6.9 ± 1.3		
**2e**, hSyn	Firing Rate	4/69	OFF	21.7 ± 1.7	paired *t*-test	t=5.17, *p*<0.001
			ON	9.9 ± 1.9		
**2f**, CaMKII	*β*-AUC power	4/4 rec.	OFF	1.5 ± 0.3 $x10^{-1}$	—*	—*
			ON	1.2 ± 0.2 $x10^{-1}$		
**2f**, hSyn	*β*-AUC power	4/4 rec.	OFF	1.5 ± 0.03 $x10^{-1}$	—*	—*
			ON	1.5 ± 0.07 $x10^{-1}$		
**2h**, CaMKII	*β*-AUC power	4/1520 opto stim	Pre-OFF	1.9 ± 1.0 $x10^{-3}$	Wilcoxon signed rank test	z=1.11, *p*=0.265
			OFF-ON	-7.5 ± 1.0 $x10^{-3}$		
**2h**, hSyn	*β*-AUC power	4/1786 opto stim	Pre-OFF	-8.8 ± 2.6 $x10^{-3}$	Wilcoxon signed rank test	z=-3.63, *p*<0.001
			OFF-ON	4.4 ± 1.9 $x10^{-3}$		
**2i**	*β*-AUC coherence	4/20 rec.	OFF	1.9 ± 0.4	paired *t*-test	t=4.25, *p*<0.001
			ON	1.6 ± 0.4		

* : Sample size too small for statistical analysis.

Supplementary Table 3. Relates to Figure 3

Figure	Parameter	n (rats/neurons)		Data Type	Data Value	Statistical test	significance level
**3c**	*β*-AUC power	6/6 rec.		OFF	1.4 ± 0.17 x10^{-1}	Wilcoxon signed rank test	z=2.20, *p*=0.031
				ON	2.8 ± 0.52 x10^{-1}		
**3d** *vs.* **3e**	*β*-phase locking	STN	28/110	Park	271.5 ± 3.8 °	Watson-Williams F test	F=339.15, *p*<0.001
			6/48	ON-ChR2	106.4 ± 9.6 °		
		Arky	6/8	Park	273.7 ± 17.5 °	—*	—*
			3/4	ON-ChR2	369.4 ± 14.9 °		
		Proto	6/17	Park	115.5 ± 17.1 °	Watson-Williams F test	F=8.97, *p*=0.0049
			5/21	ON-ChR2	163.4 ± 12.5 °		
**3f**	Firing Rate	STN	6 /48	OFF-ChR2	7.0 ± 0.8	Wilcoxon signed rank test	z=5.990, *p*<0.001
				ON-ChR2	15.0 ± 1.1	Mann-Whitney	U=1395 *p*<0.001
			28/110	Park	24.3 ± 1.1		
		Arky	3/4	OFF-ChR2	18.0 ± 4.3	—*	—*
				ON-ChR2	15.3 ± 5.4	—*	—*
			6/8	Park	12.2 ± 2.2		
		Proto	5/21	OFF-ChR2	33.7 ± 2.6	paired *t*-test	t=4.48, *p<0.001*
				ON-ChR2	41.7 ± 3.6	*t*-test	t=-4.52, *p<0.001*
			6/17	Park	20.8 ± 2.6		

* : Sample size too small for statistical analysis.

Supplementary Table 4. Relates to Figure 4

Figure	Parameter	n (rats/neurons)		Data Type	Data Value	Statistical test	significance level
**4d**	Firing Rate	Proto	5/23	OFF	22.9 ± 3.0	paired *t*-test	t=5.69, *p*<0.01
				ON	4.7 ± 2.1		
		Arky	5/3	OFF	12.6 ± 4.9	—*	—*
				ON	0.03 ± 0.03		
**4i**	Firing Rate STN	10/35		OFF	23.8 ± 2.0	Wilcoxon signed rank test	z=4.472, *p*<0.001
				ON	38.6 ± 3.5		
**4l**	*β*-AUC power	10/10		OFF	$1.2 \pm 0.14 \times 10^{-1}$	paired *t*-test	t=4.188, *p*=0.002
				ON	$0.8 \pm 0.08 \times 10^{-1}$		
**4m**	*β*-AUC power	10/3416 opto stim		Pre-OFF	$2.7 \pm 1.1 \times 10^{-3}$	Wilcoxon signed rank test	z=-27.025, *p*<0.001
				OFF-ON	$5.6 \pm 2.3 \times 10^{-3}$		

* : Sample size too small for statistical analysis.

Supplementary Table 5. Relates to Figure 5

Figure	Parameter	n (rats/neurons)	Data Type	Data Value	Statistical test	significance level
**5c**	Firing Rate	9/18	OFF-Juxta	23.5 ± 2.7	paired *t*-test	t=-7.62, *p*<0.001
			ON-Juxta	52.2 ± 5.6		
**5d**	MI	10/27	MI Arch3-GP	$3.0 \pm 0.3 \times 10^{-1}$	*t*-test	t=-1.65, *p*=0.107
		9/18	MI juxta	$3.8 \pm 0.3 \times 10^{-1}$		
**5e**	*β*-phase locking	9/18	OFF-Juxta	281.0 ± 11.6°	Watson-Williams F test	ns (F=0.29, *p*=0.593)
			ON-Juxta	274.4 ± 12.4 °		
**5i**	Firing Rate	5/27	OFF	22.2 ± 2.0	paired *t*-test	t=-3.96, *p*<0.001
			ON	37.3 ± 5.2		
**5j**	MI	10/35	MI Arch3-GP	$2.1 \pm 0.4 \times 10^{-1}$	Mann-Whitney rank sum test	ns (U=457, *p*=0.831)
		5/27	MI ChR2-STN	$1.2 \pm 0.8 \times 10^{-1}$		
**5k**	*β*-AUC power	5/5	*β*-AUC OFF	$2.2 \pm 0.4 \times 10^{-1}$	paired *t*-test	ns (t=0.919, *p*=0.41)
			β-AUC ON	$2.1 \pm 0.4 \times 10^{-1}$		
**5l**	*β*-phase locking	5/27	OFF	257.8 ± 7.1 °	Watson-Williams F test	F=4.90 *p*=0.0313
			ON	236.6 ± 7.3 °		

Supplementary Table 6. Relates to Figure 6

**Figure**	**Parameter**	**n (rats/neurons)**		**Data Type**	**Data Value**	**Statistical test**	**significance level**
**6c**	*β*-AUC power	13/13 rec.		OFF	1.2 ± 0.2 x10^{-1}	Wilcoxon signed rank test	Z=2.970, *p*=0.001
				ON	1.9 ± 0.7 x10^{-1}		
**6d** *vs.* **6e**	*β*-phase locking	STN	28/110	Park	271.5 ± 3.8 °	Watson-Williams F test	ns (F=0.763, *p*=0.50)
			13/20	ON	267.7 ± 4.1 °		
		Arky	6/8	Park	273.7 ± 17.5 °	—*	—*
			13/3	ON	70.4 ± 6.1 °		
		Proto	6/17	Park	115.5 ± 17.1 °	Watson-Williams F test	ns (F=0.830, *p*=0.367)
			13/23	ON	118.8 ± 11.3 °		
**6f**	Firing Rate	STN	13/20	OFF	7.1 ± 1.5	paired *t*-test	t=-3.50, *p*=0.002
				ON	13.6 ± 1.8	Mann-Whitney	U=486 *p*<0.001
			28/110	Park	24.3 ± 1.1		
		Arky	3/3	OFF	8.8 ± 4.3	—*	—*
				ON	7.0 ± 4.6	—*	—*
			6/8	Park	12.2 ± 2.2		
		Proto	13/23	OFF	45.5 ± 2.7	paired *t*-test	t=5.30, *p*<0.001
				ON	25.9 ± 3.0	Mann-Whitney	ns (U=151, *p*=0.229)
			6/17	Park	20.8 ± 2.6		
**6g**	MI	18/32		control	y = 0.92 – 0.05x	Linear regression	*p*<0.001, r^2=0.60
		10/27		Park	y = 0.44 – 0.006x	Linear regression	*p*=0.012, r^2=0.23

* : Sample size too small for statistical analysis.

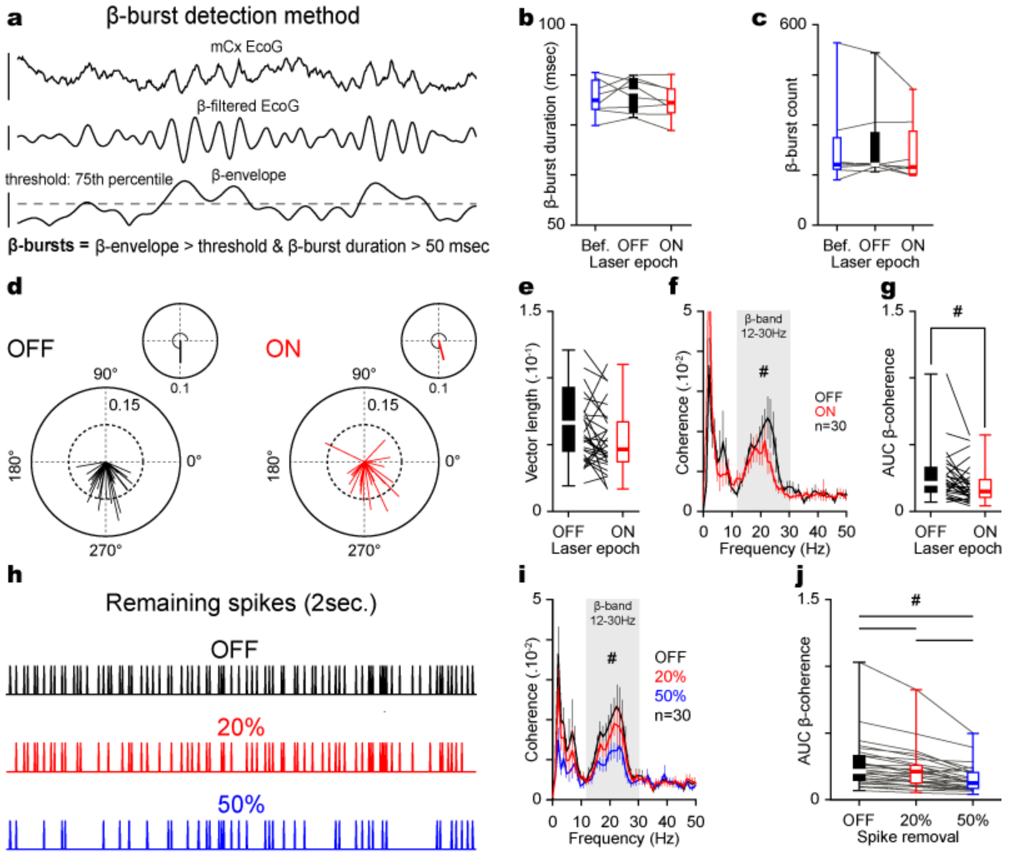

Supplementary Figure 1. Effect of mCx opto-inhibition on abnormal network dynamics in Parkinsonism. **(a)** Analytical approach used to detect the bursts of β oscillations using threshold detection method [1]. Representative example of ECoG (scales: 200 µV), β-filtered signal (12-30 Hz, scale: 50 µV), and β-envelopes (scale: 0.04 unit) with the 75th percentile threshold (dashed line). **(b-c)** Box-and-whisker plots showing the effect of mCx opto-inhibition on the β-bursts duration (**b**, Before in blue *vs.* OFF in black *vs.* ON in red, n=8, β-bursts duration Before *vs.* OFF *vs.* ON, 8.33 ± 0.17 x10^{-2} *vs.* 8.43 ± 0.16 x10^{-2} *vs.* 8.24 ± 0.16 x10^{-2} s, One Way Repeated Measures Analysis of Variance, F=1.19, p=0.33) and β-burst counts (**c**, n=8, β-bursts counts Before *vs.* OFF *vs.* ON, 231.9 ± 47.37 *vs.* 235.9 ± 43.25 *vs.* 215.6 ± 32.92 β-bursts, Friedman Repeated Measures Analysis of Variance on Ranks, X^2=0.84, p=0.65). **(d)** Individual and mean circular phase of phase-locked STN neurons during OFF and ON laser stimulation epochs (n=30, OFF *vs.* ON, 270.6 ± 6.5° *vs.* 284.5 ± 9.2°, Watson-Williams F test, F=1.42, p=0.240). **(e)** Box-and-whisker plots showing the change in vector lengths induced by mCx opto-inhibition (n=30, OFF *vs.* ON, 5.9 ± 0.4 x10^{-2} *vs.* 5.3 ± 0.4 x10^{-2}, paired t-test, t=1.70, p=0.101). **(f-g)** Mean coherence **(f)** and box-and-whisker plots of the β-band coherence **(g)** between mCx ECoG and STN unit during OFF and ON laser stimulation epochs (n=30, AUC β-band coherence OFF *vs.* ON, 2.6 ± 0.3 x10^{-1} *vs.* 2.0 ± 0.3 x10^{-1}, Wilcoxon signed rank test, z=-2.71, p=0.007). **(h)** Example of random spike removal reaching 20% (red), or 50% (blue) of the reference OFF stimulation epoch in one STN neuron (black). **(i-j)** Mean coherence **(i)** and box-and-whisker plots of the β-band coherence **(j)** between mCx ECoG and STN unit without (OFF, black) or with random spike removal (20% in red, 50% in blue, n=30, AUC β-band OFF *vs.* OFF 80% *vs.* OFF 50% *vs.* ON, 2.6 ± 0.3 x10^{-1} *vs.* 2.1 ± 0.3 x10^{-1} *vs.* 1.6 ± 0.2 x10^{-1} *vs.* 2.0 ± 0.3 x10^{-1}, Friedman repeated measures ANOVA on ranks, X^2=50.04, p<0.001, following by Dunn's post hoc test, p<0.05 for OFF *vs.* OFF 80%, OFF *vs.* OFF 50%, OFF *vs.* ON and OFF 80% *vs.* OFF 50%, q=3.9, 6.9, 4.8 and 3.0 respectively). Group data represents mean ± SEM, box-and-whisker plots indicate median, first, third quartile, min and max values.

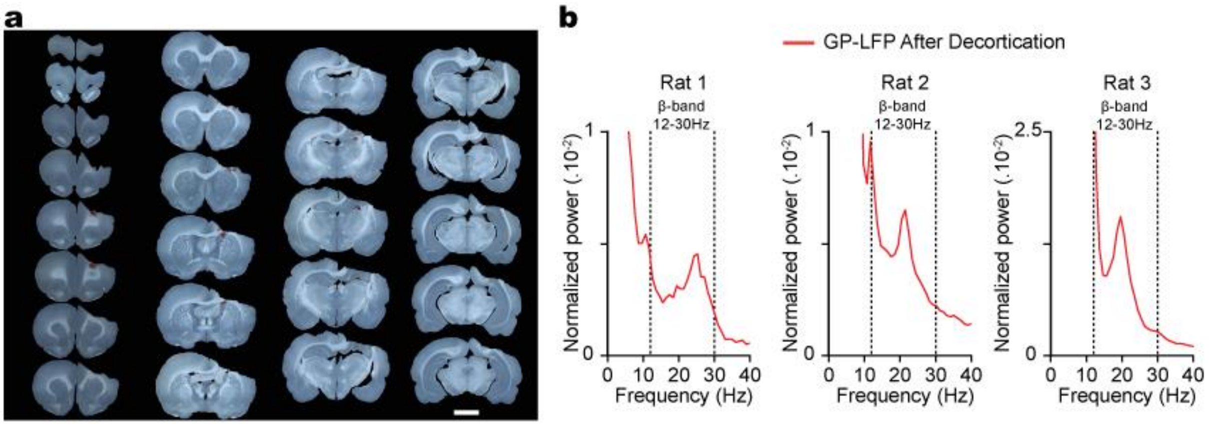

Supplementary Figure 2. Decortication experiments in 6-OHDA lesioned rats. (a) Bright-field images of coronal brain sections organized in a serial manner from rostral to caudal sections and illustrating the extend of the decortication. **(b)** Power spectrums illustrating the peak in the β frequency band (12-30 Hz) present in GP local field potentials (LFPs) recorded in 3 different 6-OHDA-lesioned rats after decortication. Although the exact generation mechanism of LFPs in non-layered structure such as basal ganglia is unclear, GP-LFPs were used here as a proxy of neuronal synchronization as previously shown during β-oscillations [2].

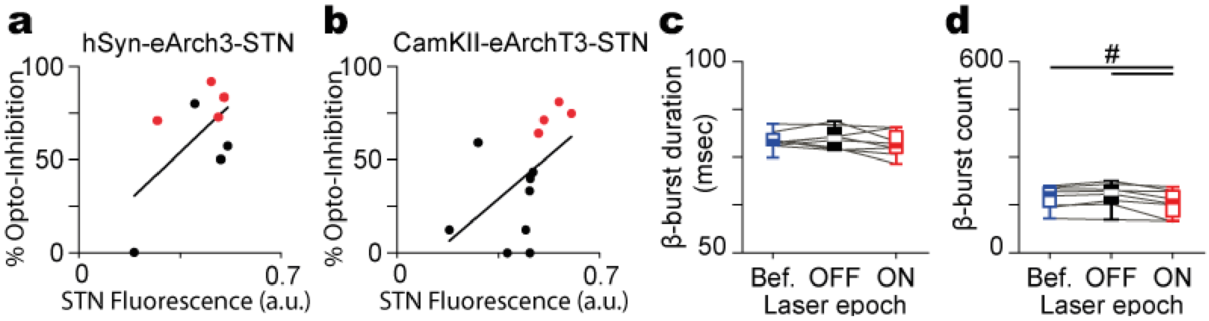

Supplementary Figure 3. Quantification of STN opto-inhibition and effect on *β* bursts dynamics. (a- b) Correlation analysis between the percentage of STN opto-inhibition determined through opto-mapping experiments and the mean EYFP fluorescence in STN for hSyn-eArch3 (**a,** linear regression r^2 = 0.3741) or CamKII-eArchT3 (**b,** linear regression r^2 = 0.2664) experiments. Red and black dots indicate the animals included or excluded from the study, respectively. Animals were discarded if they had less than 10 STN neurons recorded during opto-mapping or if the total % of STN opto-inhibition was < to 60%. **(c-d)** Box-and-whisker plots showing the effect of STN opto-inhibition on the *β*-bursts duration (**c**, n=8, *β*-burst duration Before *vs.* OFF *vs.* ON, 7.96 ± 0.07 x10^{-2} *vs.* 7.99 ± 0.1 x10^{-2} *vs.* 7.83 ± 0.1 x10^{-2} s, One Way Repeated Measures Analysis of Variance, F=1.81, *p*=0.20) and *β*-bursts counts (n=8, *β*-burst duration Before *vs.* OFF *vs.* ON, 172.75 ± 13.0 *vs.* 179.63 ± 13.9 *vs.* 157.88 ± 14.8 *β*-bursts, Friedman Repeated Measures Analysis of Variance on Ranks, F=7.19, *p*=0.007, following by Holm-Sidak post hoc test, OFF *vs.* Before, OFF *vs.* ON and Before *vs.* ON, t=1.17, 3.71 and 2.54, *p*=0.26, 0.007 and 0.047, respectively). Box-and-Whisker plots indicate median, first and third quartile, min and max values.

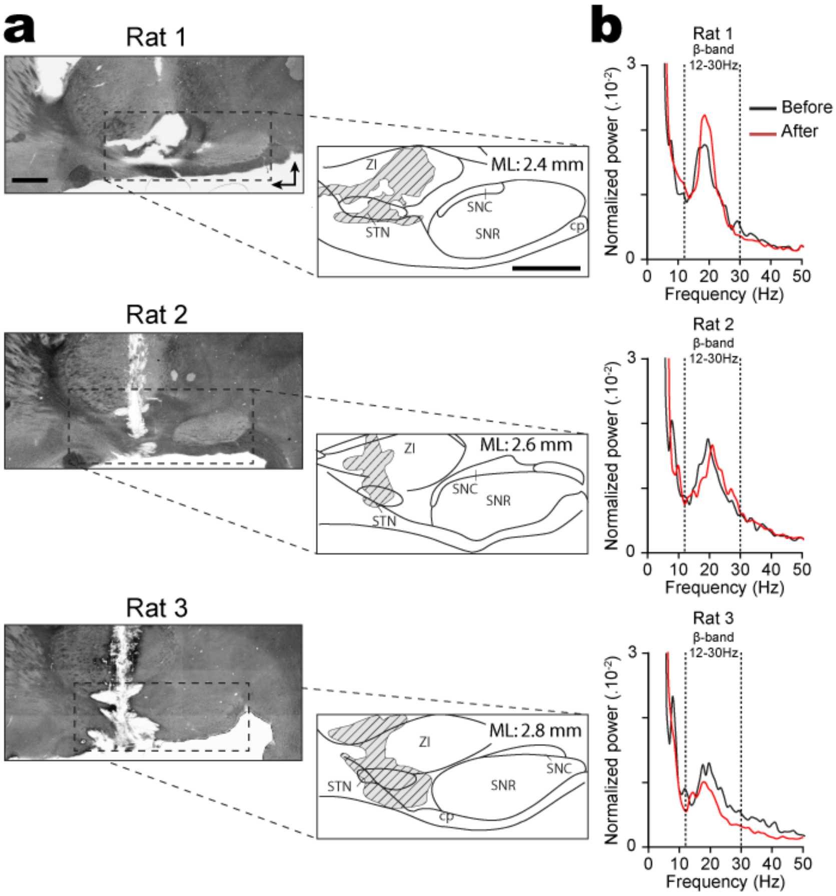

Supplementary Figure 4. Effect of STN lesion on β-oscillations expression in 6-OHDA lesioned rats. (a) Epifluorescent images showing DAPI staining (left) and schematic representation (right) of sagittal rat brain sections illustrating the extent of the STN electrolytic lesion (hashed grey area). Scale bar represent 1 mm. **(b)** Power spectrums illustrating the peak in the β frequency band (12-30 Hz) present in the ECoG before and after the full lesion of the STN in 3 different 6-OHDA-lesioned rats.

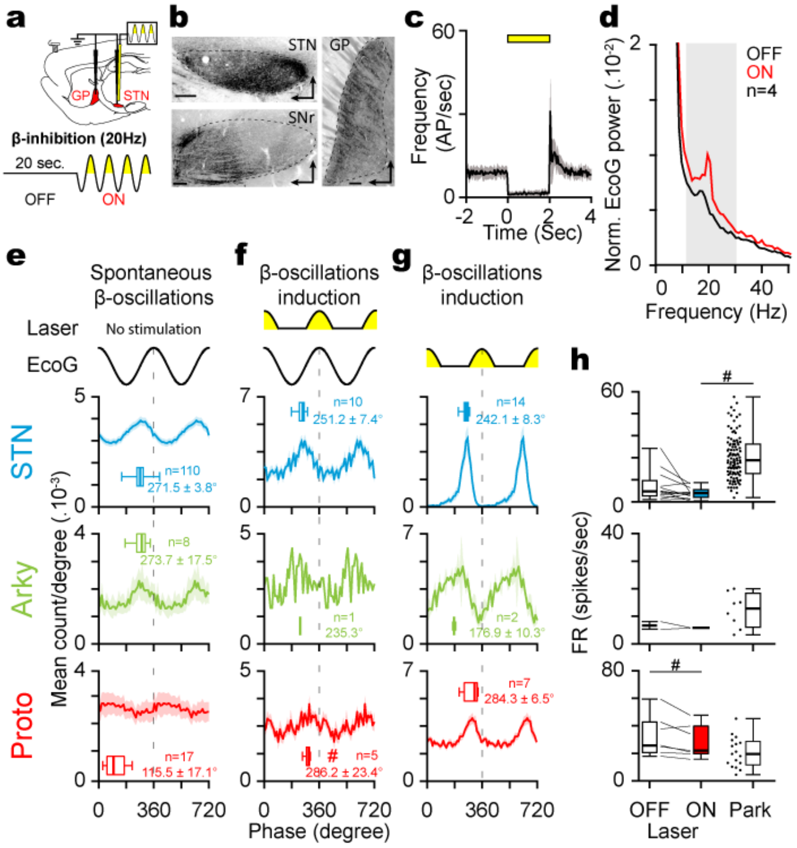

Supplementary Figure 5. STN opto-inhibitory *β*-patterning does not replicate the functional properties of parkinsonian *β*-oscillations. (a) Schematic of the eArchT3-STN experiment (top) and laser stimulation protocol (bottom) used to mimic abnormal *β*-oscillations in normal rats. **(b)** Sagittal epifluorescence images (scale: 200µm) showing a representative eArchT3-EYFP labelling in STN (top left), in SNr (bottom left) and GP (right). **(c)** PSTH of STN neurons during laser stimulations (bin: 50 ms, STN-OFF *vs.* STN-ON, n=16, 8.90 ± 2.03 *vs.* 1.37 ± 0.91 spikes/s, Wilcoxon signed rank test, z=-2.95, *p*=0.002). **(d)** Normalized power spectrum of mCx ECoG during OFF and ON laser stimulation in normal rat. **(e)** Mean phase histograms of STN (top), arkypallidal (middle), and prototypic (bottom) neurons during parkinsonian *β*-oscillations. **(f)** Mean phase histograms of STN (top), arkypallidal (middle), and prototypic (bottom) neurons induced by our opto-patterned stimulation. The mean phase value of STN neurons is not statistically different in the parkinsonian *vs.* the eArchT3 condition (STN-Park *vs.* STN-ON, n=110 *vs.* 10, 271.5 ± 3.8 ° *vs.* 251.2 ± 7.4 °, Watson-Williams test, F=2.52, *p*=0.1153), whereas the mean phase for prototypic neurons is different (Proto-Park *vs.* Proto-ON, n=17 *vs.* 5, 115.5 ± 17.1 ° *vs.* 286.2 ± 23.4 °, Watson-Williams test, F=17.74, *p*=0.0004. **(g)** Mean phase histograms of STN (top), arkypallidal (middle), and prototypic (bottom) neurons calculated from the laser peak to better illustrate the phase-relationships between STN and GP neurons**. (h)** Comparison of the change in firing rate induced by synthetic *β* as compared to parkinsonian *β*-oscillations in STN (STN-OFF *vs.* STN-ON, n=13, 8.5 ± 2.2 *vs.* 4.9 ± 0.8 spikes/sec, Wilcoxon signed rank test, Z=-1.642, *p*=0.110; STN-Park *vs.* STN-ON, n=110 *vs.* 13, 24.3 ± 1.1 *vs.* 4.9 ± 0.8 spikes/sec, Mann-Whitney rank sum test, U=160.0, *p*<0.001), arkypallidal (Arky-OFF *vs.* Arky-ON, n=2, 6.7 ± 1.4 *vs.* 5.8 ± 0.1 spikes/sec; Arky-Park *vs.* Arky-ON, n=8 *vs.* 2, 12.2 ± 2.2 *vs.* 5.8 ± 0.1 spikes/sec), and prototypic neurons (Proto-OFF *vs.* Proto-ON, n=7, 32.8 ± 5.6 *vs.* 28.7 ± 4.5 spikes/sec, paired t-test, t=2.321, *p*=0.059; Proto-Park *vs.* Proto-ON, n=17 *vs.* 7, 20.8 ± 2.7 *vs.* 28.7 ± 4.5 spikes/sec, t-test, t=2.211, *p*=0.038). Group data represents mean ± SEM, box-and-whisker plots indicate median, first, third quartile, min and max values.

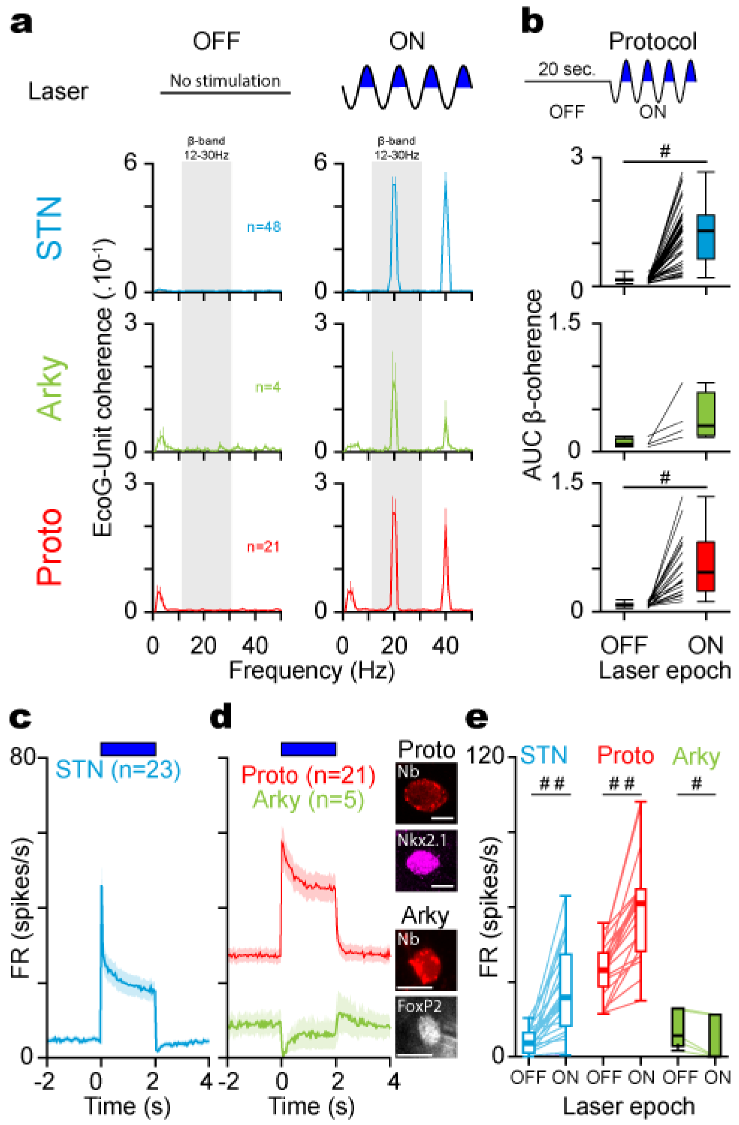

Supplementary Figure 6. Optogenetic patterning of ChR2-expressing STN neurons at β frequency. (a) Mean Coherence calculated during the OFF *vs.* ON laser stimulation between mCx ECoG and STN (blue, n=48, AUC β-coherence OFF *vs.* ON: 0.1 ± 0.007 *vs.* 1.2 ± 0.09, paired *t*-test, t=-11.90, p<0.001), arkypallidal (black, n=4, AUC β-coherence OFF *vs.* ON: 1.0 ± 0.3 *vs.* 4.0 ± 1.4 x10^{-1}), and prototypic neurons (red, n=21, AUC β-coherence OFF *vs.* ON: 0.8 ± 0.06 *vs.* 5.5 ± 0.8 x10^{-1}, paired *t*-test, t=-6.16, p<0.001). **(b)** Comparison of the β-coherence (12-30 Hz) OFF *vs.* ON laser stimulation (box-and-whisker plot, paired *t*-tests, $^{\#}$ p<0.001). **(c)** Population PSTH of ChR2-excited STN neurons in response to a 2 s laser stimulation (n=23, firing rate OFF *vs.* ON, 5.9 ± 0.9 *vs.* 26.2 ± 3.6 spikes/s, paired *t*-test, t=-6.11, p<0.001). **(d)** Population PSTH of prototypic (red, n=21, firing rate OFF *vs.* ON, 34.2 ± 2.2 *vs.* 59.4 ± 4.4 spikes/s, paired *t*-test, t=-7.85, p<0.001) and arkypallidal neurons (black, n=5, firing rate OFF *vs.* ON, 11.0 ± 3.4 *vs.* 6.8 ± 4.1 spikes/s, paired *t*-test, t=3.44, p=0.0260) in response to STN opto-excitation. Confocal fluorescence images of juxtacellularly-labelled with neurobiotin (Nb; red) and identified GP prototypic Nkx2.1+ (magenta, top) and arkypallidal FoxP2+ neurons (grey, bottom). Scale bars: 10 µm. **(e)** Box-and-whisker plots comparing the firing rate during OFF *vs.* ON STN opto-excitation stimulation (paired *t*-tests, $^{\#}$ p=0.026 and $^{\#\#}$ p<0.001). Group data represents mean ± SEM, box-and-whisker plots indicate median, first, third quartile, min and max values.

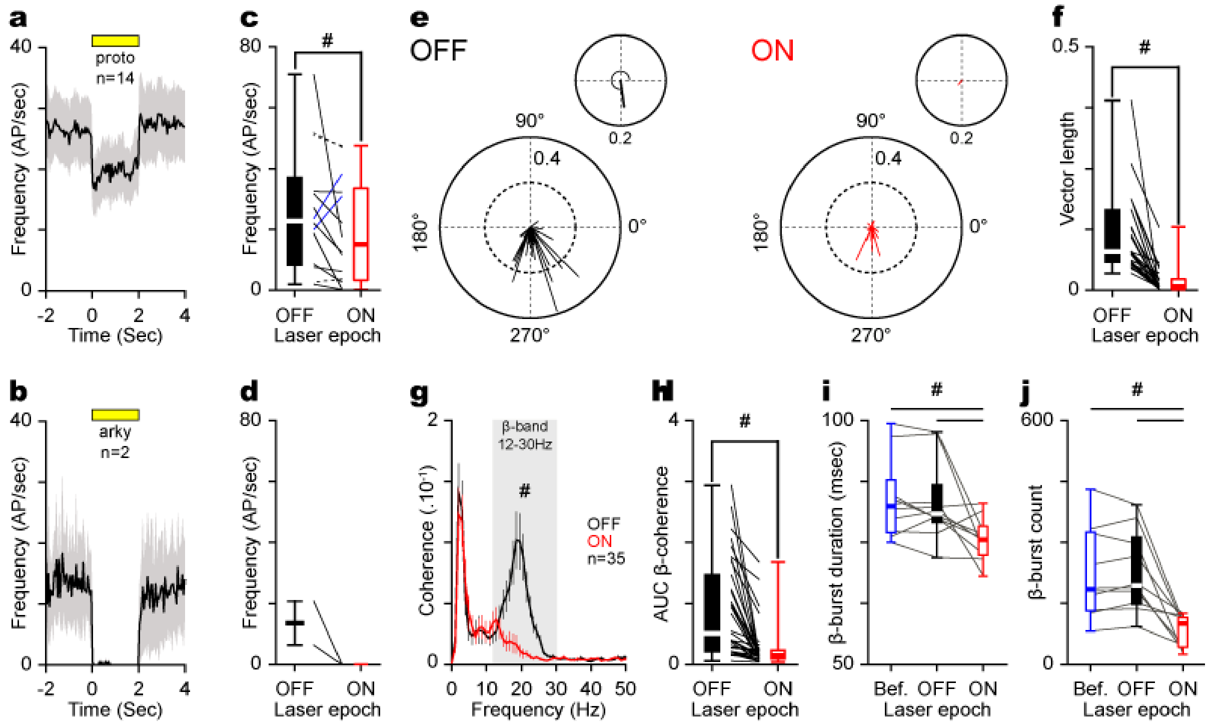

Supplementary Figure 7. Effect of GP opto-inhibition on abnormal *β*-oscillations in Parkinsonism. (a-b) Population PSTH of prototypic (n=13, firing rate OFF *vs.* ON, 26.4 ± 5.4 *vs.* 19.5 ± 4.5 spikes/s, Wilcoxon signed rank test, z=-2.93, *p*=0.068) and arkypallidal neurons (n=2, firing rate OFF *vs.* ON, 13.6 ± 7.2 *vs.* 0.08 ± 0.08 spikes/s) in response to light stimulation with optic fiber placed 2 mm above the recording electrode (data represent mean ± SEM, bin: 50 ms). **(c-d)** Box-and-whisker plots of prototypic (Wilcoxon signed rank test, *p*>0.05) and arkypallidal neurons during the OFF and ON laser stimulation. **(e)** Individual and mean circular phase of STN neurons entrained at *β* frequency during OFF and ON laser epochs (n=35, OFF *vs.* ON, 277.8 ± 6.3° *vs.* 230.0 ± 16.5°, Watson-Williams F test, F=0.510, p=0.478). **(f)** Box-and-whisker plots showing the change in vector lengths induced in STN neurons by GP opto-inhibition (n=35, OFF *vs.* ON, 1.2 ± 0.1 $x10^{-1}$ *vs.* 0.3 ± 0.06 $x10^{-1}$, Wilcoxon signed rank test, z=-5.16, *p*<0.001). **(g-h)** Mean coherence **(g)** and box-and-whisker plots of the *β*-band (12-30 Hz) coherence **(h)** between mCx ECoG and STN unit during OFF and ON laser epochs (n=35, AUC *β*-band coherence OFF *vs.* ON, 8.8 ± 1.3 $x10^{-1}$ *vs.* 2.7 ± 0.6 $x10^{-1}$, Wilcoxon signed rank test, z= -5,086, *p*<0.001). **(i-j)** Box-and-whisker plots showing the effect of GP opto-inhibition on the *β* bursts duration **(i,** n=10, β-burst duration Before *vs.* OFF *vs.* ON, 8.39 ± 0.26 $x10^{-2}$ *vs.* 8.31 ± 0.26 $x10^{-2}$ *vs.* 7.56 ± 0.14 $x10^{-2}$ s, One Way Repeated Measures Analysis of Variance, F=6.67, *p*=0.007, following by Holm-Sidak post hoc test, OFF *vs.* Before, OFF *vs.* ON and Before *vs.* ON, t=0.28, 3.01 and 3.30, *p*= 0.78, 0.015 and 0.012, respectively) and *β* bursts counts **(j,** n=10, *β*-burst duration Before *vs.* OFF *vs.* ON, 221.5 ± 34.78 *vs.* 226.70 ± 31.28 *vs.* 81.80 ± 12.41 *β*-bursts, Friedman Repeated Measures Analysis of Variance on Ranks, X^2=15.80, *p*<0.001, following by Dunn's post hoc test, OFF *vs.* Before, OFF *vs.* ON and Before *vs.* ON, q=0.89, 3.80 and 2.91, *p*>0.05, *p*<0.05 and *p*<0.05 respectively). Group data represents mean ± SEM, box-and-whisker plots indicate median, first, third quartile, min and max values.

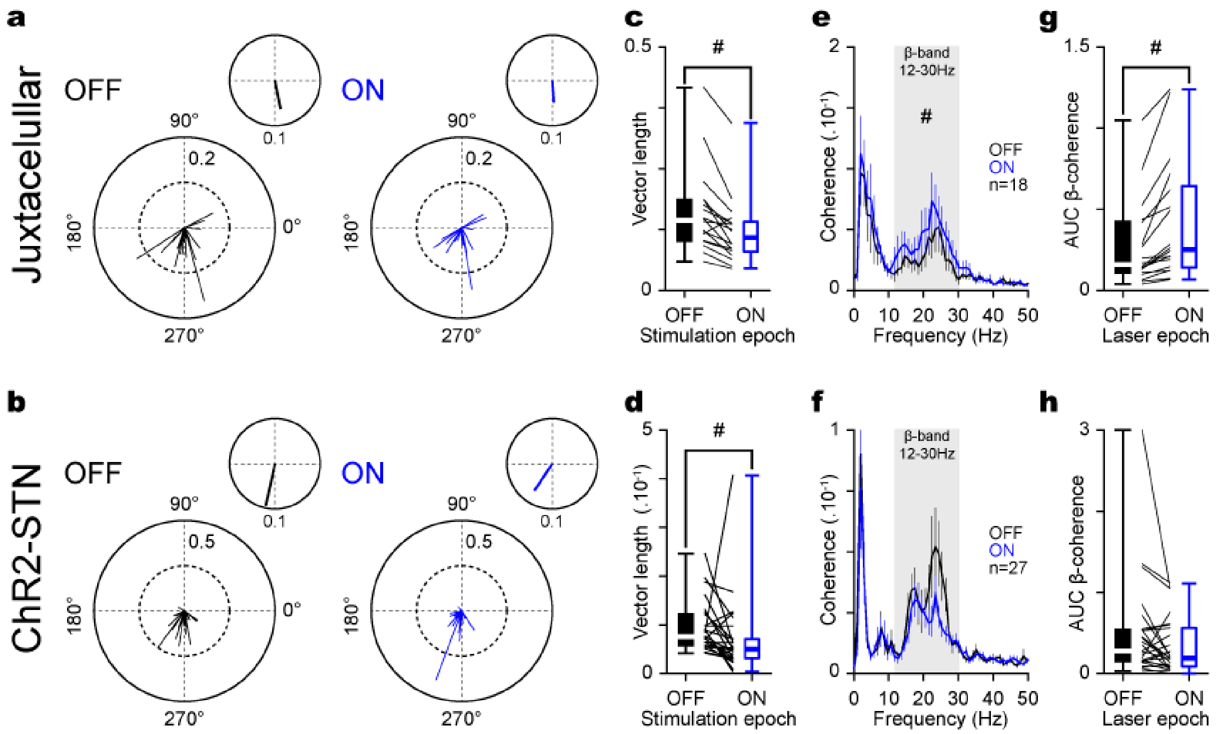

Supplementary Figure 8. Effect of STN excitation on β-synchronization. (a, b) Individual and mean circular phase of STN neurons entrained at β frequency during OFF *vs.* ON juxtacellular stimulation epochs (**a**) or the OFF *vs.* ON ChR2-STN opto-excitation (**b**). **(c, d)** Box-and-whisker plots showing the change in vector lengths induced in STN neurons by the juxtacellular excitation (**c**, n=18, mean vector length OFF-juxta *vs.* ON-Juxta, 6.4 ± 0.8 x10^{-2} *vs.* 4.9 ± 0.6 x10^{-2}, paired *t*-test, t=4.74, p<0.001) or the ChR2 opto-excitation (**d**, n=27, mean vector length OFF-ChR2 *vs.* ON-ChR2, 9.5 ± 1.0 x10^{-2} *vs.* 7.1 ± 1.5 x10^{-2}, Wilcoxon signed rank test, z=-3.123, p=0.002). **(e, f)** Mean coherence between mCx ECoG and STN units during OFF *vs.* ON juxtacellular stimulation **(e)** or the OFF *vs.* ON ChR2-STN opto-excitation **(f)**. **(g, h)** Box-and-whisker plots comparing the β-band (12-30 Hz) coherence during the OFF *vs.* ON juxtacellular stimulation (**g**, n=18 neurons, AUC β-band coherence OFF-juxta *vs.* ON-juxta, 2.7 ± 0.6 x10^{-1} *vs.* 4.0 ± 0.8 x10^{-1}, Wilcoxon signed rank test, z=3,724, p<0.001) and the OFF *vs.* ON ChR2-STN opto-excitation (**h**, n=27, AUC β-band coherence OFF-ChR2 *vs.* ON-ChR2, 5.1 ± 1.1 x10^{-1} *vs.* 3.7 ± 0.6 x10^{-1}, Wilcoxon signed rank test, z=-1.802, p=0.073). Group data represents mean ± SEM, box-and-whisker plots indicate median, first, third quartile, min and max values.

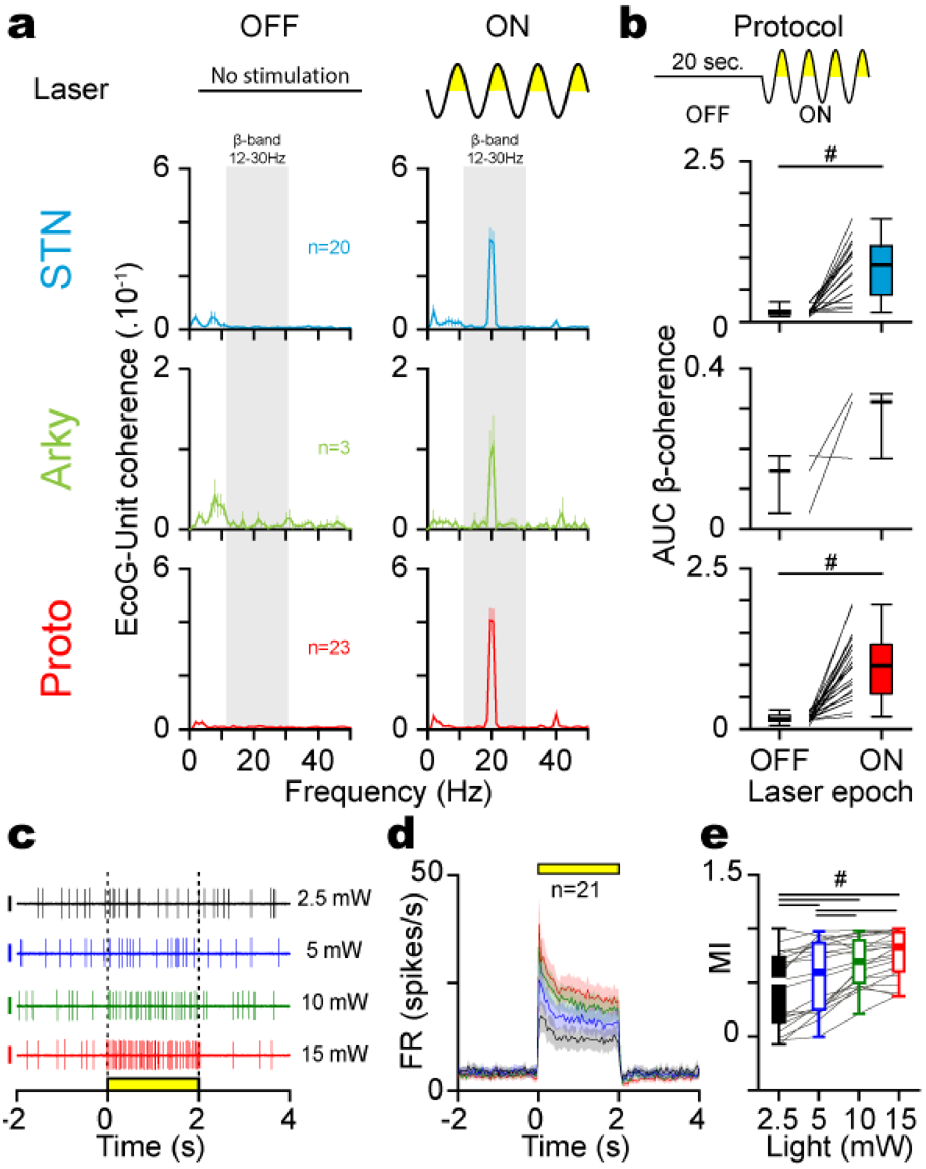

Supplementary Figure 9. Optogenetic patterning of eArch3-expressing GP neurons at *β* frequency. (a) Mean Coherence calculated during the OFF *vs.* ON laser stimulation between mCx ECoG and STN (blue), or arkypallidal (black) or prototypic neurons (red). **(b)** Box-and-whisker plots comparing the *β*-coherence (12-30 Hz) OFF *vs.* ON laser stimulation for STN (blue, n=20, AUC *β*-coherence OFF *vs.* ON: 1.6 ± 0.2 x10^{-1} *vs.* 8.3 ± 1.0 x10^{-1}, paired *t*-test, t=-6.67, p<0.001), for arkypallidal (black, n=3, AUC *β*-coherence OFF *vs.* ON: 1.2 ± 0.4 x10^{-1} *vs.* 2.8 ± 0.5 x10^{-1}), and prototypic neuros (red, n=23, AUC *β*-coherence OFF *vs.* ON: 1.8 ± 0.1 x10^{-1} *vs.* 9.7 ± 1.0 x10^{-1}, paired *t*-test, t=-7.74, p<0.001). **(c)** Example of STN firing rate disinhibition induced by increasing intensity of GP opto-inhibition (scale bar: 2 mV). **(d)** Population PSTH of STN firing to increasing intensity of GP opto-inhibition (n=21 STN neurons, GP opto-inhibition at 2.5 mW in black, 5 mW in blue, 10 mW in green, and 15 mW in red). **(e)** MI of STN neurons in response to the different intensity of GP opto-inhibition (n=21, MI at 2.5 mW *vs.* 5 mW *vs.* 10 mW *vs.* 15 mW, 4.4 ± 0.7 x10^{-1} *vs.* 5.6 ± 0.7 x10^{-1} *vs.* 6.9 ± 0.5 x10^{-1} *vs.* 7.5 ± 0.5 x10^{-1}, repeated measures ANOVA, F=34.226, p<0.001, following by Student-Newman-Keuls post hoc test, p<0.001 for 2.5 mW *vs.* 5 mW, 2.5 mW *vs.* 10 mW, 2.5 mW *vs.* 15 mW, 5 mW vs. 10 mW, 5 mW *vs.* 15 mW, q= 5.0, 10.5, 13.2, 5.5 and 8.1 respectively). Group data represents mean ± SEM, box-and-whisker plots indicate median, first, third quartile, min and max values.

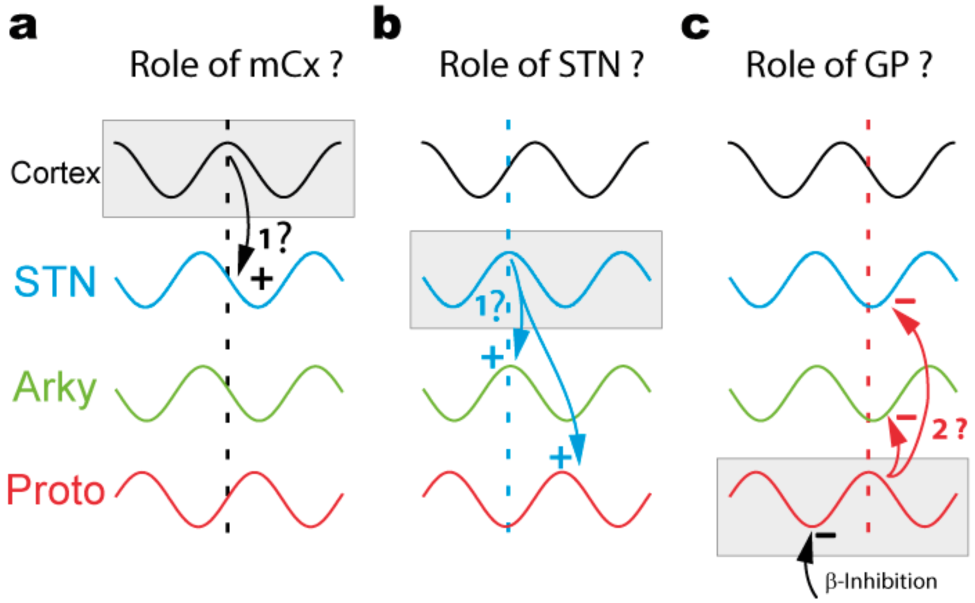

Supplementary Figure 10. Circuit mechanisms driving *β*-oscillations in parkinsonism. (a-c) Schematic representation of the neuronal *β*-oscillatory activity in cortico-basal ganglia circuits illustrating the various mechanism of *β*-oscillations generation that we have tested in this work. **(a)** The motor cortex (mCx) has been proposed to drive *β*-oscillatory activity in basal ganglia circuit, possibly through the hyperdirect pathway that directly inputs excitation at the level of the STN. We demonstrate in this work that opto-inhibition of the cortex or decortication experiments have no effect on the generation/propagation of *β*-oscillations. **(b)** Another hypothesis involves the activity of STN neurons to drive *β*-oscillatory activity in globus pallidus neurons and the rest of basal ganglia. However, we show here that STN opto-inhibition and electrolytic lesion do not affect the level of *β*-oscillations expression in these circuits. In addition, reintroduction of artificial *β*-oscillations in the STN did not reproduce the functional properties of parkinsonian *β*-oscillations. **(c)** The last hypothesis we tested in this study was the contribution of GP activity to *β*-rhythm generation/propagation. Our results indicate that *β*-activity is dependent on GP neurons and driven through inhibitory mechanisms. The inhibitory drive is likely coming from striatal indirect neurons [3] that might preferentially impact onto prototypic neurons. Our work supports the view that GP neurons, and especially prototypic neurons, are critical for the orchestration and the broadcasting of *β*-rhythm to STN and cortico-basal ganglia circuits. Abbreviations: mCx: motor cortex, STN: subthalamic nucleus, GP: globus pallidus, Arky: arkypallidal neurons, Proto: prototypic neurons.

Supplementary References

1. Cagnan, H. *et al.* Temporal evolution of beta bursts in the parkinsonian cortical and basal ganglia network. *Proc. Natl. Acad. Sci.* **116**, 16095–16104 (2019).
2. Mallet, N. *et al.* Parkinsonian beta oscillations in the external globus pallidus and their relationship with subthalamic nucleus activity. *J. Neurosci.* **28**, 14245–14258 (2008).
3. Sharott, A., Vinciati, F., Nakamura, K. C. & Magill, P. J. A population of indirect pathway striatal projection neurons is selectively entrained to parkinsonian beta oscillations. *J. Neurosci.* **37**, 0658–17 (2017).
