# Supplementary material for: The globus pallidus orchestrates abnormal network dynamics in a model of Parkinsonism
Source: Nat Commun. 2020 Mar 26;11:1570. doi: 10.1038/s41467-020-15352-3 (PMC7099038; doi:10.1038/s41467-020-15352-3)
Supplement: Supplementary file 3 — Reporting Summary [file 41467_2020_15352_MOESM3_ESM.pdf]

nature research

Corresponding author(s): nicolas Mallet

Last updated by author(s): Feb 17, 2020

Reporting Summary

Nature Research wishes to improve the reproducibility of the work that we publish. This form provides structure for consistency and transparency in reporting. For further information on Nature Research policies, see Authors & Referees and the Editorial Policy Checklist .

Statistics

For all statistical analyses, confirm that the following items are present in the figure legend, table legend, main text, or Methods section.

n/a	Confirmed	
☐	☒	The exact sample size (*n*) for each experimental group/condition, given as a discrete number and unit of measurement
☐	☒	A statement on whether measurements were taken from distinct samples or whether the same sample was measured repeatedly
☐	☒	The statistical test(s) used AND whether they are one- or two-sided<br>*Only common tests should be described solely by name; describe more complex techniques in the Methods section.*
☐	☒	A description of all covariates tested
☐	☒	A description of any assumptions or corrections, such as tests of normality and adjustment for multiple comparisons
☐	☒	A full description of the statistical parameters including central tendency (e.g. means) or other basic estimates (e.g. regression coefficient) AND variation (e.g. standard deviation) or associated estimates of uncertainty (e.g. confidence intervals)
☐	☒	For null hypothesis testing, the test statistic (e.g. *F*, *t*, *r*) with confidence intervals, effect sizes, degrees of freedom and *P* value noted<br>*Give P values as exact values whenever suitable.*
☒	☐	For Bayesian analysis, information on the choice of priors and Markov chain Monte Carlo settings
☐	☒	For hierarchical and complex designs, identification of the appropriate level for tests and full reporting of outcomes
☐	☒	Estimates of effect sizes (e.g. Cohen's *d*, Pearson's *r*), indicating how they were calculated

Our web collection on statistics for biologists contains articles on many of the points above.

Software and code

Policy information about availability of computer code

Data collection	The collection of electrophysiological data was performed with Spike2 software version 8. The collection of histological images was performed using Axio Imager 2 (Zeiss) for epifluorescence microscopy or the Las X 3.6 software (Leica) for confocal microscopy. Additional details are provided in methods section.
Data analysis	Data analyses of electrophysiological data was achieved with custom script written in Spike2 v8 or Matlab R2016a. Statistical analyses were performed using SigmaPlot 12 (Systat Software). Data analyses of the fluorescence images was performed using Fiji (ImageJ 1.52i).Additional details are provided in methods section. The codes for the analysis used in this study are available from the corresponding author upon reasonable request.

For manuscripts utilizing custom algorithms or software that are central to the research but not yet described in published literature, software must be made available to editors/reviewers. We strongly encourage code deposition in a community repository (e.g. GitHub). See the Nature Research guidelines for submitting code & software for further information.

Data

Policy information about availability of data

All manuscripts must include a data availability statement. This statement should provide the following information, where applicable:

- Accession codes, unique identifiers, or web links for publicly available datasets
- A list of figures that have associated raw data
- A description of any restrictions on data availability

The raw data that support the findings are available from the corresponding author upon reasonable request.

Field-specific reporting

Please select the one below that is the best fit for your research. If you are not sure, read the appropriate sections before making your selection.

[x] Life sciences [ ] Behavioural & social sciences [ ] Ecological, evolutionary & environmental sciences

For a reference copy of the document with all sections, see nature.com/documents/nr-reporting-summary-flat.pdf

Life sciences study design

All studies must disclose on these points even when the disclosure is negative.

Sample size	The number of animals were determined taking into account the expected effect size, the confidence interval, the margin of error we considered acceptable in each experiments. The probability to falsely reject the null hypothesis was set to alpha=0.05, z1=1.96. All statistical analyses were two-tailed statistical tests (see methods section 'Statistical analysis'). The precise number of animals for each experiment are provided in the supplementary tables.
Data exclusions	The animals that did not reach sufficient level of virus transfection as determined by optogenetic mapping (that is 60% of neurons manipulating by the light stimulation, see methods section 'functional optogenetic mapping') and histological verification (as determined by ROI of the EYFP signal, see methods section 'Tissue processing and histological control') were excluded from the study (see exact numbers provided in the methods section). Proving the correctness of negative results (i.e. in our case the absence of light effect on measured parameter X) requires careful considerations. Indeed, because 'the absence of effect is not a proof of absence' and to avoid misinterpreting our results, we decided to discard the animals that did not reach the best level of viral infection (as determined through optogenetic mapping and histological verifications). This logic forms the rational of our data exclusion.
Replication	Each experiments were repeated so that our data are based on at least three independent experiments with similar results. The precise number of repeats are provided in the figure legend and supplemental table.
Randomization	I am sorry for the misinterpretation here. The animals were indeed arbitrarily chosen when forming the experimental groups upon virus injection in order to avoid accidental bias.
Blinding	The same experimenters performed both the surgeries and the electrophysiological recordings so they could not be blind to the experiments. However, all our analyses were performed using computational codes that perform the same digital signal analyses to all group allocation or experimental conditions.

Reporting for specific materials, systems and methods

We require information from authors about some types of materials, experimental systems and methods used in many studies. Here, indicate whether each material, system or method listed is relevant to your study. If you are not sure if a list item applies to your research, read the appropriate section before selecting a response.

Materials & experimental systems

n/a	Involved in the study	
[ ]	[x]	Antibodies
[x]	[ ]	Eukaryotic cell lines
[x]	[ ]	Palaeontology
[ ]	[x]	Animals and other organisms
[x]	[ ]	Human research participants
[x]	[ ]	Clinical data

Methods

n/a	Involved in the study	
[x]	[ ]	ChIP-seq
[x]	[ ]	Flow cytometry
[x]	[ ]	MRI-based neuroimaging

Antibodies

Antibodies used	Chicken anti-GFP antibody, Aves Labs, Cat#GFP-1020; RRID:AB_10000240<br>Rabbit anti-Nkx2.1 (TTF-1) antibody, Santa Cruz Biotechnology, Cat#sc-13040; RRID:AB_793532<br>Goat anti-FoxP2 antibody, Santa Cruz Biotechnology, Cat#sc-21069; RRID:AB_2107124<br>Alexa Fluor 488 AffiniPure Donkey Anti-Chicken, Jackson ImmunoResearch Labs, Cat# 703-545-155, RRID:AB_2340375<br>CY™5-conjugated Donkey Anti-Rabbit, Jackson ImmunoResearch Labs, Cat# 711-175-152, RRID:AB_2340607<br>Brilliant Violet™ 421-conjugated Donkey Anti-Goat, Jackson ImmunoResearch Labs, Cat# 705-675-147, RRID:AB_2651102
Validation	All antibodies used in this study have been validated by the manufacturer through both western-blot and immunohistochemistry. In addition, these antibodies have been appropriately characterized (based on immunohistological staining and unbiased stereological cell counting) in previous publication (see ref. 39). Accordingly, we have added this reference to the corresponding methods section.

Animals and other organisms

Policy information about studies involving animals; ARRIVE guidelines recommended for reporting animal research

Laboratory animals	For this study we used adults rats, Sprague Dawley males aged from 9 to 21 weeks. All animals were obtain from authorized suppliers (Janvier Labs).
Wild animals	No wild animals were used in this study
Field-collected samples	No fied-collected samples were used in this study
Ethics oversight	This project was approved by the French ministry of higher education and research and the ethical committee of CNRS, Aquitaine Region (accreditation number 5012079-A)

Note that full information on the approval of the study protocol must also be provided in the manuscript.
